# Reduced alpha power predicts recurrence risk in major depressive disorder

**DOI:** 10.1016/j.ynirp.2026.100357

**Published:** 2026-05-22

**Authors:** Amy Tong, Roland Zahn, Jennifer A. Gethin, Wael El-Deredy, Jason Palmer, Gráinne McLoughlin

**Affiliations:** a Institute of Psychiatry, Psychology & Neuroscience, Department of Psychological Medicine, Centre for Affective Disorders, King's College London, London, SE5 8AF, UK; b National Service for Affective Disorders, South London and Maudsley NHS Foundation Trust, London, SE5 8AZ, UK; c The University of Manchester and Manchester Academic Health Sciences Centre, School of Psychological Sciences, Neuroscience and Aphasia Research Unit, Manchester, M13 9PL, UK; d Interdisciplinary Center of Biomedical and Engineering Research for Health, Universidad de Valparaíso, Chile; e School of Mathematical and Data Sciences, West Virginia University, Morgantown, WV, United States; f Social, Genetic and Developmental Psychiatry Centre, King's College London, De Crespigny Park, London, SE5 8AF, UK

**Keywords:** EEG, Depression, MDD, Recurrence, Alpha, Frontal alpha asymmetry

## Abstract

**Background:**

A high proportion of individuals with major depressive disorder (MDD) develop recurrent episodes. A better understanding and the identification of markers of recurrence risk are needed to develop prophylactic treatments and to support treatment decisions. Electroencephalography (EEG) is a scalable approach to identifying potential markers. Alpha band (8-13Hz) power and frontal alpha asymmetry (FAA) have been implicated in the pathophysiology of depression, however prospective studies of recurrence risk are sparse. Here, we investigated frontal alpha power and FAA in remitted MDD patients, to assess their potential as predictors of future depressive episodes.

**Methods:**

In total, 84 participants (n = 53 medication-free remitted MDD, n = 31 control participants with no family history of MDD) completed resting EEG testing. Remitted MDD patients were followed up clinically to determine recurrence over 14 months. Resting frontal alpha power and FAA were compared between groups (controls, stable remission, subclinical symptoms, recurrent episode) and binary logistic regression models were used to predict recurrence risk in remitted MDD patients.

**Results:**

Patients who developed a recurring episode showed reduced frontal alpha power at baseline compared to controls and patients who remained in stable remission. No group effect was found for FAA. When combining clinical predictors with frontal alpha power, prediction accuracy for recurring episodes was 88%.

**Conclusions:**

This study demonstrates that reduced frontal alpha power has the potential to be further developed as a marker for recurrence risk in MDD, and could contribute to recurrence risk prediction models. This calls for larger studies using cross-validated prediction modelling techniques.

## 1 Introduction

The high burden of major depressive disorder (MDD) is due not only to its high lifetime prevalence (Hasin et al., 2018; Arias De La Torre et al., 2021), but also its recurring nature (Greden, 2001), with the likelihood of future episodes increasing to 90% after three lifetime episodes (Eaton et al., 2008). Recurrent depression contributes substantially to global disability, highlighting the need for research into mechanisms that drive recurrence. Identifying these mechanisms is a key step towards a longer-term aim to develop treatments which reduce risk of recurrence and improve long-term outcomes. While clinical factors, such as the number of prior episodes and residual symptoms are associated with recurrence risk (Buckman et al., 2018), these markers only account for a modest proportion of prognostic variance (Lawrence et al., 2021). Electroencephalography (EEG) is a method that allows for investigation of neurophysiological vulnerability to recurrence, with the advantage that it has a high temporal resolution and is widely deployable and scalable, so that it could be used clinically to stratify patients. Identifying electrophysiological features of recurrence risk provides insights into the neural processes due to the close relationship between EEG and neural network function.

Studies of EEG spectral band power have consistently implicated alpha-band oscillations (8-13Hz) in depression (de Aguiar Neto and Garcia Rosa, 2019). Alpha activity is hypothesized to play a role in attention through selectively inhibiting task-irrelevant cortical regions, and optimizing the timing of neuronal activation in task-relevant regions (Klimesch, 2012). Two measures of alpha that have been studied in MDD are alpha power and asymmetry in alpha power across frontal electrode sites, termed “frontal alpha asymmetry”.

Studies of alpha power in MDD have largely focussed on current MDD, with power in the alpha band having the best discriminating capability when classifying participants as either those with MDD or healthy controls (Hosseinifard et al., 2013). The direction of alpha power differences remains inconsistent, with some studies reporting decreased alpha power in MDD patients compared to controls (Lee et al., 2018), and others reporting increases (Kemp et al., 2010). Contradicting results may reflect differences in EEG setup, and demographics of study samples, such as age, sex, and medication, all of which may affect alpha power (Almeida Montes et al., 2015; Tröndle et al., 2023; Cave and Barry, 2021).

The few studies that have examined alpha in remitted, rather than current, MDD patients have overall found remitted MDD patients to have reduced alpha power compared to controls (Almeida Montes et al., 2015; Čukić et al., 2020; Suzuki et al., 1996). As approximately 50% of remitted patients experience a recurrence within 14 months (Viguera et al., 1998), a group of remitted MDD participants will contain both those who will have another episode, and those who will remain stable. From cross-sectional studies, it is unknown whether decreased alpha power reflects a scarring-effect of past depressive episodes, or a trait-like vulnerability to future episodes. The ability to differentiate these two groups is necessary to knowing if altered alpha power may precede depressive episodes, or if reduced alpha power is a marker of lifetime MDD. To elucidate this, prospective studies are needed.

Studies dating back to the 1980s have found MDD patients to have increased frontal alpha asymmetry (FAA) - i.e. increased alpha power at left vs. right frontal electrodes, compared with control participants (Kołodziej et al., 2021). A small number of studies has confirmed these findings in remitted depression (Stewart et al., 2010; Gotlib, 1998; Henriques and Davidson, 1990). FAA was proposed to reflect that withdrawal states, such as sadness and fear, are associated with increased activity in right frontal cortical areas, and therefore decreased right frontal alpha power (Coan et al., 2001; Davidson et al., 2002). Conversely, approach states, such as anger or joy were hypothesized to relate to increased cortical activity in left frontal areas. However, four meta-analyses have failed to confirm the robustness of FAA as a marker of depression (Kołodziej et al., 2021; van der Vinne et al., 2017; Thibodeau et al., 2006; Luo et al., 2025). A few studies of never-depressed individuals have found increased FAA to predict a first episode of depression within 3 years (Nusslock et al., 2011), or increased severity of depressive symptoms over 12 months (Stewart and Allen, 2018; Mitchell and Pössel, 2012), showing that FAA may be a marker for primary vulnerability. Only one study has prospectively investigated FAA in remitted depression: Berwian et al. found that FAA differences between sad and neutral clips predicted relapse of depression following anti-depressant withdrawal (Berwian et al., 2024). It is not known if FAA would also predict recurrence of depression in medication-free remitted MDD patients.

Uncertainty remains as to the generators of alpha activity measured at frontal electrodes. Due to volume conduction, EEG activity recorded at the scalp does not necessarily originate from nearby regions. Attempts to identify the generators of frontal electrode alpha EEG activity in MDD have correlated EEG alpha measures with current density (the flow of electric current per unit area), as calculated by tomography methods which provide three-dimensional estimates of current density from EEG power, rather than localising activity specifically measured at frontal electrodes. Saletu et al. found a negative correlation between Hamilton Depression Scores and both FAA and alpha current density localised to the left pre-frontal cortex (Saletu et al., 2010). Smith et al., however, found that frontal EEG asymmetry correlated with current density at the dorsal anterior cingulate cortex and medial-lateral regions of the frontal lobe (Smith et al., 2018). Further research is necessary to reconcile conflicting results and clarify the contributions of different brain regions to frontal alpha EEG power.

In this prospective study, we investigated whether EEG alpha band power at frontal electrodes predicts recurrence of depression in remitted MDD patients. Medication-free patients were followed-up for 14 months after baseline EEG data collection. Measures of interest were frontal alpha power and frontal alpha asymmetry. The predictive potential of using frontal alpha power in conjunction with clinical predictors at the individual level was assessed through logistic regression models. We further completed exploratory source analyses to identify the origin of frontal EEG alpha measures.

## 2 Methods and materials

### 2.1 Participants

Participants with remitted MDD and control participants with no personal or family history of mood disorders or schizophrenia were recruited as part of the BlameDepredict project, a prospective study to investigate recurrence risk (NHS ethics reference: 07/H1003/194).

**Fig. 1:**
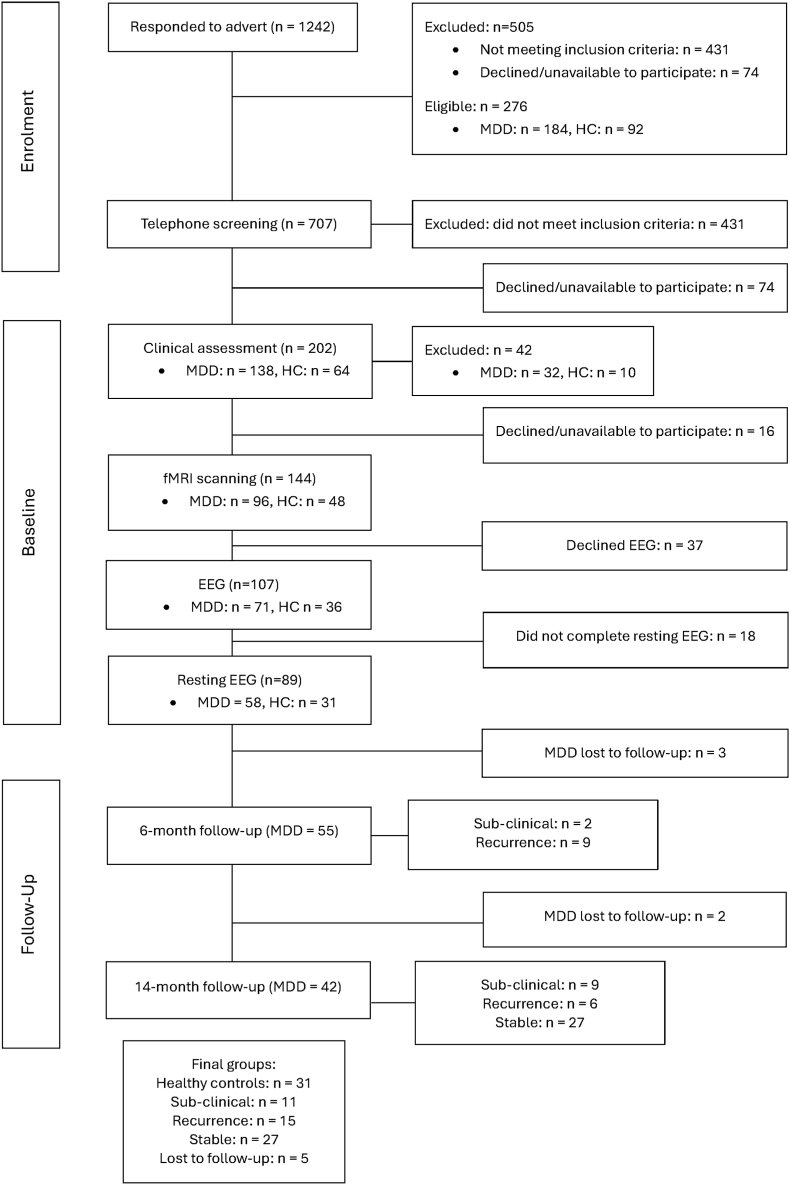
CONSORT diagram showing recruitment and study participation at each stage.

After responding to print and online advertisements, potential participants attended a telephone screening interview. Eligible participants were then clinically assessed to determine inclusion into the study, and to collect baseline clinical and behavioural data. The number of participants included/excluded at each stage are detailed in Fig. 1. Participants gave informed consent verbally for the telephone interview and written for other study visits. Compensation was given for their participation and travel costs.

Inclusion criteria for remitted MDD participants were: MDD diagnosis according to the Structured Clinical Interview I (SCID-I) for DSM-V-TR (American Psychiatric Association, 2000), being in remission for ≥6 months. Exclusion criteria included current Axis I disorders, and past comorbid Axis I disorders being the likely cause of depressive symptoms (see Supplemental Methods for full exclusion criteria). Remitted MDD and control participants were psychotropic-medication-free and right-handed. Participants had normal or corrected-to-normal vision and were native English speakers, due to study tasks with text-based stimuli.

Following the clinical assessment, 96 MDD and 48 control participants completed tasks probing social emotions during fMRI scanning. Previous papers have described clinical, fMRI, and behavioural results (Lythe et al., 2015). A subgroup of 107 participants took part in EEG testing, which included an adapted version of the social emotion task; the results of which are not discussed in the present paper. Of the subgroup, 18 participants did not complete resting-state testing, as it was added after the study began. The final sample for the present study was 58 remitted MDD and 31 control participants. Remitted MDD participants remained in the study for up to 14 months, during which they had follow-up telephone assessments at 3 months, and face-to-face clinical visits (also carried out on the phone if participants were unable to attend in-person) at 6 and 14 months to detect possible recurrent major depressive episode (MDEs). Study participation ended if an MDE was detected. 27 participants remained in stable remission, 15 had a recurring episode, and 11 developed significant MDD symptoms without meeting full diagnostic criteria (subclinical group). Five participants were lost to follow-up.

### 2.2 Clinical assessment

In the baseline clinical assessment, symptoms were assessed by study investigators using the mood and anxiety modules from the SCID-I. Investigators had received training and inter-rater reliability was excellent (Zahn et al., 2015). Findings from the clinical assessment were also confirmed by a senior psychiatrist (RZ). Measures also included the Beck Depression Inventory II (BDI-II) (Beck et al., 1988), Montgomery-Åsberg Depression Rating Scale (MADRS) (Montgomery and Asberg, 1979), and Global Assessment of Functioning Scale (GAF) (Endicott et al., 1976).

During follow-up appointments, the well-validated Longitudinal Interval Follow-up Evaluation interview for DSM-IV (LIFE) (Keller et al., 1987) was used to assess MDE symptoms and psychosocial functioning with excellent inter-rater reliability (Lythe et al., 2015). The LIFE-IV uses the Psychiatric Status Rating (PSR) score to classify MDE status: no symptoms = 1, mild symptoms causing no relevant impairment/distress = 2, mild symptoms that cause no more than moderate impairment/distress = 3, major symptoms not meeting full MDE criteria = 4, symptoms meeting full MDE criteria = 5, most severe forms of MDE = 6. Based on participants’ worst PSR score during the follow-up period, they were assigned to groups: 1) *Stable* remission [PSR 1-3 and not requiring treatment], 2) *Subclinical* symptoms [PSR = 3 and requiring treatment or PSR = 4], 3) *Recurring* episode [PSR = 5-6].

### 2.3 EEG acquisition

EEG was recorded after the baseline clinical visit, with a mean delay of 54 days (SD = 29.8) for the remitted MDD group, and 69 days (SD = 78.2) for the control group. The clinical status of participants was re-assessed for relevant changes on the day of EEG recording. EEG was acquired using a 64-electrode ActiveTwo system and Actiview software (BioSemi) at a sampling rate of 512 Hz, using the 10-20 International System electrode placement (Gethin, 2015). Participants completed 2 min each of eye-closed and eyes-open resting state periods. Only eyes-closed data have been analysed presently, following previous studies which investigated spectral band power in MDD (Smith et al., 2017), and due to its improved reliability compared to an eyes-open acquisition (Metzen et al., 2022).

### 2.4 EEG data pre-processing

EEG data preprocessing and analysis were carried out using EEGLAB v2024.1 (Delorme and Makeig, 2004) and MATLAB (Inc. TM, 2023). The data were down-sampled to 256 Hz and filtered using a .5-30 Hz band pass filter. Bad EEG channels were removed using the Clean Rawdata EEGLAB plugin (Kothe et al., 2019). Independent component analysis (ICA) using the Picard algorithm (Ablin et al., 2018) was applied to each participant's data. ICA weights were calculated on a copy of the data filtered to 1-30 Hz, with high amplitude segments removed, to ensure a high quality ICA decomposition (Winkler et al., 2015). ICA weights were then transferred to the .5-30 Hz data. Eye components identified by ICLabel (Pion-Tonachini et al., 2019) (probability threshold = .8), were removed. The data were re-referenced to the average of all channels, and the Clean Rawdata plug-in was used for automated artifact rejection. Current source density (CSD) was then calculated for each electrode using CSD Toolbox (Kayser and Tenke, 2006) due to findings that CSD transformations improve detection of FAA effects by reducing contribution of non-frontal sources to frontal alpha (Smith et al., 2017). Only F3 and F4 were required for the primary analysis – however all bad channels were interpolated for the purposes of creating descriptive topographical plots. See Supplemental Methods for additional EEG processing methods.

### 2.5 EEG measures

Alpha (8-13Hz) and theta (4-8Hz) power for electrode sites F3 and F4 were calculated using the spectopo() function, using Welch's method (Welch, 1967) to calculate power spectral density. Power measures were natural log-transformed, as is standard practice when analysing frontal alpha asymmetry (Smith et al., 2017).

K-means clustering was used to identify brain sources of activity projecting to F3 and F4. For full EEG methods, see Supplemental Methods.

### 2.6 Data analysis

A primary repeated-measures ANOVA was conducted to examine the effects of laterality (within-subject factor of F3 vs. F4) and group (between-subjects factor) on alpha power. This approach allowed investigation of both absolute band power differences and frontal alpha asymmetry.

Two secondary ANOVA models were run to evaluate the specificity of observed group effects to the alpha band, compared to the theta band. The theta band was chosen for comparison because studies have found that theta is second to alpha in its power to discriminate between MDD participants and controls (Hosseinifard et al., 2013).

Logistic regressions were used to evaluate the ability of frontal alpha power, in combination with known clinical factors, to predict individual risk of recurring MDE compared to remaining in stable remission. The primary model included F3/F4 average alpha power, and as in our previous fMRI-based prediction model (Lawrence et al., 2021): baseline BDI-II score, and the number of MDEs (categorized into nonrecurrent [i.e., 1 previous episode], recurrent [2–4 episodes], and highly recurrent [≥5 episodes]). To minimize the number of predictors in light of the small sample size (n = 46), only these clinical measures were used as predictors, as they were included as a-priori predictors based on previous wider literature (discussed in (Lawrence et al., 2021)). Because of our sample size below n = 50, we were unable to meaningfully use doubly-nested cross-validated regularized logistic regression models previously developed for predicting recurrence risk (Lawrence et al., 2021). Two supplementary models were also tested: one including sex as an additional predictor, and the other replacing alpha power with theta power. All statistical analyses were conducted using SPSS (version 29.0.2.0) with an alpha level of .05 2-tailed.

## 3 Results

### 3.1 Group characteristics

The four groups (stable, recurring, subclinical, and control) were compared for key demographic and clinical measures (Table 1). There were no differences in age, education, or MADRS between any of the groups. As expected, the recurring group had a higher baseline BDI-II score than all other groups. The stable and recurring groups had lower GAF scores than the control group.

**Table 1:** Demographic characteristics and symptoms.

Variable	Remitted MDD (n = 53)									
	Stable Group		Sub-Clinical Group		Recurring Group		Control Group			
	n = 27		n = 11		n = 15		n = 31			
	n	%	n	%	n	%	n	%	χ^2^ (3)	p

Sex									3.612	.306
Female	18	67	10	91	9	60	19	39		
Male	9	33	1	9	6	40	12	61		

	M	SD	M	SD	M	SD	M	SD	F(3, 80)	p

Age	40.63	12.329	40.09	10.502	38.67	14.13	35.7	15.006	.734	.535
Years of Education	17.59	2.099	17.09	2.809	16.07	2.434	17.4	2.169	1.546	.209
BDI-II Score	2.48	3.043	3.36	4.456	6.13	4.868	1.06	1.861	8.102	<.001^a^
MADRS Score	.89	1.502	.55	.934	1.33	1.952	.71	1.321	.797	.499
GAF Score	86.85	4.504	87.45	4.367	84.27	5.922	89.2	2.499	4.36	.003^a^

BDI-II, The Beck Depression Inventory II; MADRS, The Montgomery–Åsberg Depression Rating Scale; GAF, Global Assessment of Functioning.

^a^Significant group difference at an uncorrected two-sided threshold of p ≤ .05.

### 3.2 Group-based EEG results

**Fig. 2:**
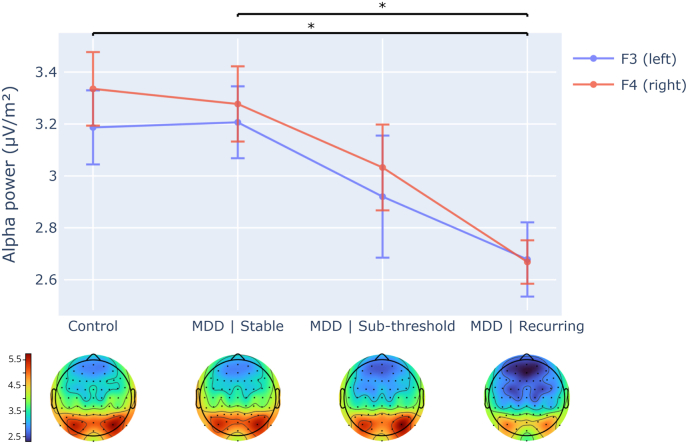
Alpha power (8-13Hz) differences between clinical groups (primary analysis) at F3 (left) and F4 (right) frontal electrode sites. Errors bars show ±1 standard error. Alpha power was calculated using CSD-transformed data. Topographical scalp maps depict the distribution of alpha power for all sites for each group, using the same CSD-transformed alpha power measure plotted for F3 and F4; color bars indicate alpha power values. A significant group effect was found for mean values of alpha power at frontal electrodes (mean of F3 and F4) for control and major depressive disorder (MDD) groups (see Table 2). ∗ between-groups post-hoc tests significant at p ≤ .05, 2-sided, uncorrected.

There was a main group effect for alpha power at F3 and F4 (Table 2). Post-hoc analyses showed that alpha power at F3 and F4 was lower for the recurring group compared to the control group and the stable remission group (Fig. 2, Table 2). Alpha power for the sub-clinical group was not statistically different to any other groups. Alpha power was greater at F4 than F3 across groups (Table 2). Importantly, no group-by-site interaction was found, indicating there was no difference in alpha asymmetry between groups.

**Table 2:** Group-based EEG results.

Band	Site(s)	Control	Stable	Sub-clinical	Recurring	Group			Site			Group-by-Site		
		(n = 31)	(n = 27)	(n = 11)	(n = 15)	F(3, 80)	partial η^2^	p	F(1, 83)	partial η^2^	p	F(3, 80)	partial η^2^	p
Alpha	F3	3.19 (.795)	3.21 (.719)	2.92 (.780)	2.68 (.554)	2.742	.093	.049∗˟	4.624	.055	.035∗	.918	.033	.436
	F4	3.34 (.789)	3.28 (.753)	3.03 (.549)	2.67 (.716)									
Theta	F3	3.66 (.621)	3.57 (.669)	3.36 (.792)	3.40 (.703)	1.180	.042	.322	1.797	.022	.184	.861	.031	.465
	F4	3.78 (.665)	3.63 (.695)	3.39 (.729)	3.38 (.785)									
						Group			Band			Group-by-Band		
						F(3, 80)	partial η^2^	p	F(1, 83)	partial η^2^	p	F(3, 80)	partial η^2^	p

Alpha	F3/F4 avg.	3.26 (.777)	3.24 (.723)	2.98 (.653)	2.67 (.613)	1.960	.068	.127	95.535	.544	<.001∗	2.515	.086	.064
Theta	F3/F4 avg.	3.72 (.624)	3.60 (.674)	3.38 (.744)	3.39 (.726)									

Results of repeated measures analyses of variance (ANOVA) probing the effect of group and electrode site on alpha power (primary analysis) and theta power (secondary analysis), as well as the effect of group and band on average power at F3 and F4.

∗ = significant at p = .05 (uncorrected), 2-sided.

˟Post-hoc analyses of group differences in alpha power at F3 and F4 found control > recurring (MD = .588 (SE = .226), 95% CI [.139, 1.04], p = .011), and stable > recurring (MD = .569 (SE = .231), 95% CI [.109, 1.03], p = .016).

Exploratory analyses found no correlation between average alpha power at F3 and F4 and BDI-II (r = −.080, *p=*.178) or GAF score (r = .148, *p* = .178), confirming that group power differences were not due to differing residual symptom levels between groups.

To test if the group effect was specific to the alpha band, rather than extending to theta, a repeated-measures ANOVA was conducted to investigate the effect of frequency band (alpha vs. theta) and group on averaged EEG power at F3 and F4. A band-by-group interaction approached significance, indicating a potential group-dependent difference in power between the alpha and theta band (Table 2, Fig. S1). There was a main effect of band, with theta power being greater than alpha power across the groups. There was no effect of group.

**Fig. 3:**
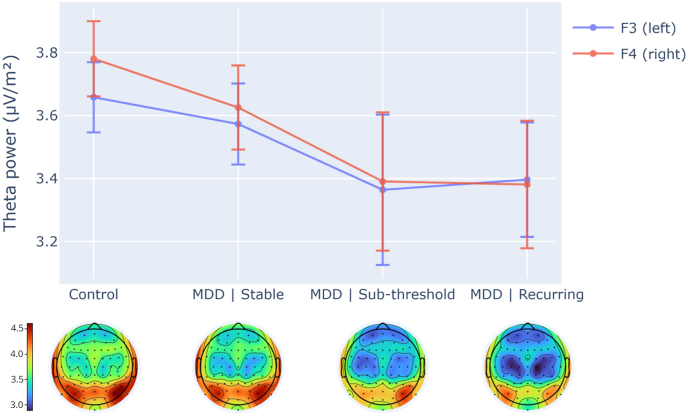
Theta power (4-8Hz) differences between groups (supporting analysis) at F3 (left) and F4 (right) frontal electrode sites. Errors bars show ±1 standard error. Theta power was calculated using CSD-transformed data. Topographical scalp maps depict the distribution of theta power for all sites for each group, using the same CSD-transformed theta power measure plotted for F3 and F4. No main effects were found (see Table 2).

An additional repeated measures ANOVAs were conducted for F3 and F4 power in the theta band (4-8Hz). There was no significant effects of group, hemisphere, or group-by-hemisphere interaction (Fig. 3, Table 2). Results from sex-effect tests are reported in Supplementary Results.

### 3.3 Prediction modelling

**Fig. 4:**
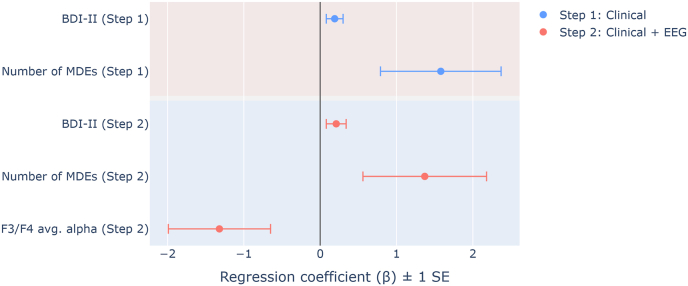
Logistic model coefficients (β) for associations between clinical predictors and frontal EEG alpha power (F3 and F4 average), and MDD recurrence. Step 1 of the model included Beck Depression Inventory II scores (BDI-II) and the number of previous major depressive episodes (MDEs), correctly classifying 78.6% of cases. Adding frontal alpha power in the second step significantly increased classification accuracy to 88.1%, explaining 47.7% of the variance (Nagelkerke R^2^).

A stepwise logistic regression was conducted to evaluate whether frontal alpha power could improve prediction of recurring MDE risk, compared to clinical variables alone. In the first step, using only BDI-II scores and the number of MDEs, the model was statistically significant, correctly classifying 78.6% of cases and explaining 37% of the variance (Nagelkerke R^2^). Adding F3/F4 average alpha power in the second step significantly improved the model (p = .029), increasing classification accuracy to 88.1% and explaining 47.7% of the variance (Nagelkerke R^2^). Notably, this step increased sensitivity to 80% from 47% - however, with large confidence intervals. Lower alpha power, higher baseline BDI-II scores, and a greater number of prior MDEs were associated with an increased likelihood of developing a recurring episode over 14 months (Table 3, Fig. 4). The order of steps was reversed for a secondary model to evaluate the predictive ability of frontal alpha power without clinical factors. In the first step, using only average alpha power at F3 and F4 as a predictor, the model was statistically significant, χ^2^ (1) = 6.46, p = .011, correctly classifying 66.7% of cases and explaining 19.6% of the variance (Nagelkerke R^2^). See Supplemental Results for full reporting of secondary models.

**Table 3:** Binary logistic regression for predicting stable vs. recurring episode (n = 44).

		Model parameters					Model step		Overall model				
		*β*	SE	Wald	*p*	Odds Ratio [95% CI]	χ^2^	*p*	% correct	Sensitivity (%) [95% CI]	Specificity (%) [95% CI]	PPV	NPV
Step 1	BDI-II Score	.19	.11	3.27	.071	1.21 [.98 1.49]	13.19	.001	78.6	46.67 [21.27 73.41]	96.30 [81.03 99.91]	87.50	76.47
	Number of MDEs	1.58	.79	4.05	.044	4.88 [1.04 22.83]							
Step 2	BDI-II Score	.21	.13	2.84	.092	1.24 [.97 1.59]	4.74	.029	88.1	80.00 [75.71 99.09]	92.59 [75.71 99.09]	85.71	89.29
	Number of MDEs	1.37	.81	2.85	.091	3.95 [.80 19.49]							
	F3/F4 avg. alpha	−1.32	.67	3.95	.047	.27 [.07 .98]							

∗ significant at *p* < .05 threshold, two-tailed. BDI-II, The Beck Depression Inventory II; Number of MDEs categorized into nonrecurrent [i.e., 1 previous episode], recurrent [2–4 episodes], and highly recurrent [≥5 episodes]. SE, standard error; CI, confidence interval; PPV, positive predictive value; NPV, negative predictive value.

## 4 Discussion

In this prospective study, we found that reduced alpha power in medication-free remitted MDD patients predicted depressive episode recurrence within 14 months. Individuals at risk of recurrence had lower alpha power at frontal electrode sites compared to both those who remained in stable remission, and controls with no personal or family history of depression. There was no difference in frontal alpha power between the stable remission group and the control group. Using frontal alpha power improved prediction of recurrent episodes compared to using clinical measures predictors alone. We found no differences in frontal alpha asymmetry across any of the remitted MDD or healthy control groups.

The current study, to our knowledge, is the only prospective study, to investigate resting alpha power in the prediction of recurrence risk in depression. Our finding that individuals who experienced a recurrent depressive episode over 14 months had reduced alpha power extends cross-sectional studies of remitted depression. Cucik et al. (Čukić et al., 2020) found reduced high alpha power (10-12 Hz) in right prefrontal sites, decreased low alpha power (8-10 Hz) in central and posterior sites, but increased low alpha in right lateral frontal and prefrontal sites in remitted and current depression. We focused our analysis on the frontal electrodes F3 and F4 to probe both alpha power and frontal alpha asymmetry and to limit the number of comparisons. The variation in electrode sites showing reduced alpha power across studies may reflect methodological differences, particularly our use of a current source density transformation to reduce volume conduction, enhance spatial specificity (Tenke and Kayser, 2012), and to make our results comparable to the existing frontal alpha asymmetry literature (Smith et al., 2017).

Decreased levels of alpha in those at risk of recurrence may reflect heightened cortical and autonomic arousal. Alpha power has been shown to negatively correlate with electrodermal skin conductance level, a measure of autonomic arousal (Barry et al., 2020), and reductions in alpha power were observed in tasks designed to increase arousal, such as pain induction (Rho et al., 2023). Furthermore, lower heart rate variability, indicative of reduced parasympathetic control and a dominance of the sympathetic nervous system, has been reported in individuals with remitted depression compared to controls (Bassett et al., 2016; Dell'Acqua et al., 2021). A partial correlation has also been found between heart rate variability and resting-state EEG alpha power (Kawashima et al., 2024). Taken together, these findings suggest that reduced alpha power may serve as a neurophysiological marker of hyperarousal in those at risk for recurrent depression.

Our previous study used structural and functional MRI data to improve individualised prediction of recurrence risk compared to using clinical markers, using machine and statistical learning methods (Lawrence et al., 2021). In the current study, we built upon these findings by showing that EEG measures also improve predictions using clinical markers alone. Due to the small sample size in this study, we used a logistic regression model, rather than a statistical/machine learning-based approach. We found clinical markers to have a higher classification accuracy (79%) compared to our past study (69%), which may be a result of this particular subsample and specific predictors used, and cannot be used as an accurate estimate. Results are in keeping with the wider literature confirming baseline BDI-II and number of previous MDEs as the most reproducible for predicting recurrence risk in MDD (Hardeveld et al., 2010; Kessing et al., 2004; Ruhe et al., 2019). The addition of frontal electrode alpha power increased prediction of recurrence risk by 10%, suggesting the potential value of combining neurophysiological data with clinical information. While alterations in alpha power are not specific to MDD (Ippolito et al., 2022), our work shows that EEG-derived measures appear to capture aspects of vulnerability to recurrence that are not fully accounted for by clinical markers alone. Further studies with larger sample sizes and cross-validation are needed to confirm these findings, and could incorporate other EEG measures (e.g. signal complexity) which are altered in MDD (de Aguiar Neto and Garcia Rosa, 2019).

Importantly, we found no differences in FAA between the groups, contradicting previous findings of greater left versus right frontal alpha power in medication-free remitted depression (Stewart et al., 2010; Gotlib, 1998; Henriques and Davidson, 1990). This discrepancy may be due to differences in sample characteristics. A meta-analysis of FAA studies in MDD found a three-way interaction of age-by-symptom severity-by-gender (van der Vinne et al., 2017). Previous studies showing FAA effects in remitted depression often used younger, university-aged participants (Stewart et al., 2014), whereas the current study had a mean age of 35-40 across groups. Additionally, uncontrolled factors like caffeine consumption may have impacted FAA measurements (Stewart et al., 2011). Frontal alpha asymmetry has been proposed as a marker for a trait vulnerability to depression. However, our findings do not support its utility as a marker for recurrence risk specifically, especially as frontal alpha asymmetry may be influenced by a complex interplay of different measures.

Stronger frontal alpha asymmetry differences may be elicited by using emotional challenge tasks, rather than a resting-state. In a study from Stewart et al., controls and participants with current or past MDD made emotional facial expressions of approach emotions (happy and angry) and withdrawal emotions (afraid and sad). For the emotional expression conditions, the researchers found FAA differences between both MDD groups and controls, for all EEG reference montages tested, but not for the resting-state condition. In another study, Berwian et al. found that sad vs neutral movie clips asymmetry was a predictor for relapse of depression following antidepressant withdrawal (Berwian et al., 2024). Asymmetry effects may therefore be more robust against methodological differences if measured in an emotional state. Our ongoing study in a larger replication sample (Zahn et al., 2022), will address this by using an emotion induction task, designed to elicit feelings of blame and praise towards oneself and others, during EEG recording.

Our exploratory source localization analyses indicated that alpha power projecting to frontal electrodes do not reflect frontal brain sources. Rather, sources had their peak localization within the left dorsal anterior cingulate cortex (dACC), the striatum, and left globus pallidus (see Supplemental Results). The dACC, which is associated with the modulation of attention and motivational processes (Bush et al., 2000) has been implicated in imaging studies of MDD. Resting-state functional connectivity of the dACC and other limbic regions has been shown to be altered in MDD (Schwartz et al., 2019; Crowther et al., 2015; Taylor et al., 2021), and functional connectivity between the dACC and parahippocampal gyrus predicted severity of MDD symptoms in one study (Crowther et al., 2015).​ As alpha power was proposed to facilitate communication between different brain regions by synchronising cycles of excitability (Clayton et al., 2018), reduced alpha in those vulnerable to MDD recurrence may reflect disrupted communication in emotion-relevant networks which is also in keeping with the event-feature-emotion complex model of adaptive moral emotions (Moll et al., 2005).

These findings extend work by Almeida Montes et al., who found reduced alpha current density in medication-naïve MDD patients in the ACC among other areas. Those who remitted following fluoxetine treatment, had increased levels of alpha current density in the left-orbital cortex, basal ganglia, and hippocampus prior to treatment, compared to non-remitters. Patients who had received fluoxetine for 1 year had lower alpha current density than the controls both prior to treatment and during remission. At a group level, this suggests that reduced alpha activity is a trait marker of vulnerability to depression and of difficult-to-treat depression, rather than being related to a depressed state. Our results advance this by demonstrating that more severely reduced alpha specifically predicts vulnerability to depression recurrence.

On a more cautionary note, we had a modest sample size, which may cause overestimation of effect sizes, may have led to false negative results, and prevented us from using cross-validated prediction modelling techniques. Future studies in larger samples are therefore needed to replicate our findings. In addition, further studies of neural sources of alpha power are required, particularly given the limitations of EEG source analysis methods (Bigdely-Shamlo et al., 2013), including variations in brain geometry and, in this study, the absence of electrode position co-registration to individual structural brain images.

## 5 Conclusions

Our findings demonstrate that reduced alpha power could serve as a marker for recurrence risk in depression and may reflect an asymptomatic state of major depressive disorder with ongoing need for interventions to prevent recurrence risk. We found no differences in frontal alpha power between healthy controls and stable remitted MDD participants, showing that reduced alpha power is specifically associated with the pathophysiology of recurrence risk in MDD. We also demonstrated that frontal electrode alpha power can improve the prediction of an individual's likelihood to experience a recurring depressive episode, compared to clinical measures alone. Due to the scalability of EEG and the minimal setup required, frontal electrode alpha power has the potential for use in clinical settings to support decision-making processes and could serve as a treatment target for recurrent depression such as using EEG-based neurofeedback studies. This calls for larger future studies to replicate our finding using cross-validated regularized regression models which have the potential to pave the way for novel scalable biomarkers and neurofeedback treatments for depression.

## Financial disclosures

RZ is a private psychiatrist service provider at The London Depression Institute and co-investigator on a Livanova-funded observational study of Vagus Nerve Stimulation for Depression. RZ has received honoraria for talks at medical symposia sponsored by Lundbeck as well as Janssen. Prof Zahn has collaborated with EMOTRA, EMIS PLC and Depsee Ltd. RZ is affiliated with the D’Or Institute of Research and Education, Rio de Janeiro and advises the Scients Institute, USA. AT, GM, JAG, and WED have no potential conflicts of interest.

## Funding sources

This work was funded by an MRC clinician scientist fellowship (G0902304) to RZ. JAG was funded by an EPSRC PhD studentship. RZ was funded by UK Medical Research Council grants (MR/T017538/1 & MR/Y008545/1) and National Institute for Health and Care Research (NIHR) Biomedical Research Centre at South London and Maudsley NHS Foundation Trust and King's College London. The views expressed are those of the authors and not necessarily those of the NHS, the NIHR or the Department of Health and Social Care. AT was funded by the Medical Research Council Doctoral Training Partnership (Ref: MR/N013700/1). AT is associated with the International Research Training Group (IRTG) 2773 “Risks and Pathomechanisms of Affective Disorders”, funded by the Deutsche Forschungsgemeinschaft (DFG, German Research Foundation) (GRK2773/1- 454245598). GM is funded by the Tianqiao and Chrissy Chen Institute and the UK Medical Research Council (UKRI539). WED acknowledges the support of ANID Chile: FONDECYT 1241695 and Exploracion 13240064.

## CRediT authorship contribution statement

**Amy Tong:** Conceptualization, Data curation, Formal analysis, Funding acquisition, Methodology, Visualization, Writing – original draft. **Roland Zahn:** Conceptualization, Funding acquisition, Investigation, Methodology, Project administration, Resources, Supervision, Writing – review & editing. **Jennifer A. Gethin:** Conceptualization, Data curation, Funding acquisition, Investigation, Methodology, Project administration, Writing – review & editing. **Wael El-Deredy:** Conceptualization, Investigation, Methodology, Resources, Software, Supervision, Writing – review & editing. **Jason Palmer:** Methodology, Software, Writing – review & editing. **Gráinne McLoughlin:** Conceptualization, Methodology, Resources, Supervision, Writing – review & editing.

## Declaration of competing interest

The authors declare that they have no known competing financial interests or personal relationships that could have appeared to influence the work reported in this paper.

## Data Availability

Data will be made available on request.
