# Supplementary material for: Reduced alpha power predicts recurrence risk in major depressive disorder
Source: Neuroimage Rep. 2026 May 22;6(2):100357. doi: 10.1016/j.ynirp.2026.100357 (PMC13224359; doi:10.1016/j.ynirp.2026.100357)

# Reduced alpha power predicts recurrence risk in major depressive disorder

SUPPLEMENTARY INFORMATION

# Supplemental Methods

## Full exclusion criteria

Further to the exclusion criteria listed in the main manuscript, participants were excluded if they met any of the following: MRI contraindications, currently taking psychotropic medication or having psychotherapy, significant psychosocial impairment as an indicator of a possible personality disorder (assessed on the Global Assessment of Functioning scale (GAF), Montgomery Åsberg Depression Rating Scale (MADRS) score of > 10, current self-harming behaviour, developmental disorders, learning disabilities, Addenbrooke’s Cognitive Exam-R score < 88 (completed in participants >50 years old), or neurological or physical illnesses that significantly alter brain function or blood flow.

## EEG processing

To ensure a high-quality independent component analysis (ICA) decomposition, an amplitude-based artifact rejection method to remove extreme outliers in the EEG signal was applied prior to the ICA. First, the mean and standard deviation were computed for the maximum absolute amplitude across all channels for each time point. To identify artifacts, a rejection threshold was set at 5 standard deviations above the mean maximum amplitude. To prevent edge effects and ensure complete removal of artifacts, one second of data was rejected surrounding each identified artifact.

Alpha (8-13Hz) and theta (4-8Hz) power were computed at 0.5 Hz intervals across each frequency band, using the current source density (CSD) transformed data, and the values were averaged to obtain a single power estimate per band. Two-second windows were used with one-second overlaps. All participants met the criteria of at least 20 two-second clean segments for inclusion in the power spectrum density calculation. The mean number of two-second segments used per participant was 101 (SD=26.9). Following pre-processing, the average length of clean data for each participant was 114 seconds (SD=20.6s).

## Secondary prediction models

In addition to the primary prediction model reported in the main manuscript, two secondary logistic models were run. The first model reversed the stepwise order of the primary model, entering average F3/F4 power as the sole predictor in the first step, in order to evaluate its ability to predict depression recurrence independently of clinical variables. The second model extended the primary model by adding an additional step that included sex as a predictor, given that men exhibited lower alpha power compared to women.

## Source analysis

Components identified by ICLabel as brain components with 80% likelihood were clustered using a K-means clustering algorithm. Independent components with dipoles located within the brain and with less than 15% residual variance between estimated dipole projection and IC scalp topography were clustered based on spectral features, dipole locations, and scalp topography. Principal component analysis was used to reduce the dimensionality of spectral features and scalp topographies to 10 and 15 for clustering, respectively. Seven clusters were calculated and outlier components further than three standard deviations from any of the cluster centroids were removed.

Three clusters were selected based on having high levels of alpha power and projection to electrode sites F3 and F4. Two participants did not have components included in any of the three clusters – see Supplementary Table S1 for the number of participants and ICs included in each cluster.

# Supplemental Results

## Sex effects

The potential influence of sex on alpha power was examined using a repeated-measures ANOVA with factors of sex and laterality. Group effects on alpha power were reevaluated after controlling for sex to ensure robustness. Controlling for group, men had reduced alpha power at F3 and F4, compared to women, see Supplementary Table S1 and Supplementary Figure S2. There was a trend laterality-by-sex interaction (p = .054), suggesting a potential sex difference in frontal alpha asymmetry, with men tending towards reduced FAA (i.e. women had increased left relative to right frontal alpha power than men). The significant group effect on alpha power remained when controlling for sex (F(1,3) = 3.222, p = .027).

## Secondary prediction models

A stepwise logistic regression was conducted to evaluate whether frontal alpha power could independently predict the risk of recurring MDEs without clinical variables. In the first step, using only average alpha power at F3 and F4 as a predictor, the model was statistically significant, correctly classifying 66.7% of cases and explaining 19.6% of the variance (Nagelkerke R²).

An additional model was conducted to assess whether adding sex as a predictor improved classification accuracy, given that men exhibited lower alpha power than women. When using the same predictors as the primary model, the model was statistically significant, correctly classifying 88.1% of cases and explaining 47.7% of the variance (Nagelkerke R²). In the third step, adding sex as a predictor did not significantly improve the model (p=.570), reducing classification accuracy to 85.7 % while slightly increasing the explained variance to 48.4% of the variance (Nagelkerke R²; see Supplementary Table S4). This demonstrates that while there are sex differences in alpha power, they do not meaningfully contribute to predicting recurrence risk beyond clinical and EEG predictors.

## Source analysis

The centroids of the three selected clusters were located in the left dorsal anterior cingulate cortex (Brodmann Area 24), the striatum, and left globus pallidus (Supplementary Table S2, Supplementary Figure S3).

# Supplementary Tables

**Supplementary Table S1.** Results of repeated measures ANOVAs probing the effect of sex on alpha power at F3 and F4.

| Sex | Site(s) | Control | Stable | Sub-clinical | Recurring |  | Sex | | | |  | | Laterality | | | |  | | Laterality-by-sex | | |
| --- | --- | --- | --- | --- | --- | --- | --- | --- | --- | --- | --- | --- | --- | --- | --- | --- | --- | --- | --- | --- | --- |
|  |  |  |  |  |  |  | F(1, 76) | partial η² | p |  | | F(1, 76) | | partial η² | p |  | | F(1, 76) | | partial η² | p |
| Female |  | (n = 19) | (n = 18) | (n = 10) | (n = 9) |  | 4.568 | .057 | .036* |  | | 7.229 | | .087 | .009 |  | | 3.819 | | .048 | .054 |
|  | F3 | 3.40 (.731) | 3.25 (.724) | 3.02 (.753) | 2.93 (.553) |  |  |  |  |  | |  |  |  |  |  | |  |  |  |  |
|  | F4 | 3.46 (.786) | 3.31 (.819) | 3.09 (.546) | 2.88 (.741) |  |  |  |  |  | |  |  |  |  |  | |  |  |  |  |
| Male |  | (n = 12) | (n = 9) | (n = 1) | (n = 6) |  |  |  |  |  | |  |  |  |  |  | |  |  |  |  |
|  | F3 | 2.85 (.804) | 3.13 (.746) | 1.97 (-) | 2.30 (.278) |  |  |  |  |  | |  |  |  |  |  | |  |  |  |  |
|  | F4 | 3.13 (.780) | 3.21 (.638) | 2.48 (-) | 2.35 (.596) |  |  |  |  |  | |  |  |  |  |  | |  |  |  |  |
| * = significant at p = .05 (uncorrected), 2-sided | | | | | | | | | | | | | | | | | | | | | |

**Supplementary Table S2.** Number of participants and independent components included in each cluster. Locations of cluster centroids given in Montreal Neurological Institute space.

| Cluster | Participants | ICs | MNI Coordinates |
| --- | --- | --- | --- |
| L-ventral ACC | 61 | 135 | [-7 -7 47] |
| Striatum | 61 | 134 | [27 -11 13] |
| L-Globus Pallidus | 66 | 117 | [-22 -4 -2] |

**Supplementary Table S3**. Logistic regression of stable vs. recurring episode (n = 44), with F3/F4 avg. power as the only predictor in the first model step.

|  |  | Model Parameters | | | | |  | Model step | |  | Overall model | | | | |
| --- | --- | --- | --- | --- | --- | --- | --- | --- | --- | --- | --- | --- | --- | --- | --- |
|  |  | *β* | SE | Wald | *p* | Odds Ratio [95% CI] |  | χ² | *p* |  | % correct | Sensitivity (%) [95% CI] | Specificity (%)  [95% CI] | PPV | NPV |
| Step 1 | F3/F4 avg. alpha | -1.27 | .55 | 5.26 | .022 | .28 [.10 .83] |  | 6.46 | .011 |  | 66.7 | 54.44 [23.38 83.25] | 70.97 [51.96 85.78] | 40.00 | 81.48 |
| Step 2 | F3/F4 avg. alpha | -1.32 | .13 | 2.84 | .047 | .27 [.07 .98] |  | 11.47 | .003 |  | 88.1 | 80.00 [51.91 95.67] | 92.59 [75.71 99.09] | 85.71 | 89.29 |
|  | BDI-II Score | .21 | .81 | 2.85 | .092 | 1.24 [.97 1.59] |  |  |  |  |  |  |  |  |  |
|  | Number of MDEs | 1.37 | .248 | .004 | .091 | 3.95 [.80 19.49] |  |  |  |  |  |  |  |  |  |
| * significant at p < .05 threshold, two-tailed. BDI-II, The Beck Depression Inventory II; Number of MDEs categorized into nonrecurrent [i.e., 1 previous episode], recurrent [2–4 episodes], and highly recurrent [≥5 episodes]. SE, standard error; CI, confidence interval; PPV, positive predictive value; NPV, negative predictive value. | | | | | | | | | | | | | | | |

**Supplementary Table S4**. Logistic regression of stable vs. recurring episode (n = 44), adding sex as a predictor.

|  |  | Model parameters | | | | |  | Model step | |  | Overall model | | | | |
| --- | --- | --- | --- | --- | --- | --- | --- | --- | --- | --- | --- | --- | --- | --- | --- |
|  |  | *β* | SE | Wald | *p* | Odds Ratio [95% CI] |  | χ² | *p* |  | % correct | Sensitivity (%) [95% CI] | Specificity (%) [95% CI] | PPV | NPV |
| Step 1 | BDI-II Score | .19 | .11 | 3.27 | .071 | 1.21 [.98 1.49] |  | 13.19 | .001 |  | 78.6 | 46.67  [21.27 73.41] | 96.30 [81.03 99.91] | 87.50 | 76.47 |
|  | Number of MDEs | 1.58 | .79 | 4.05 | .044 | 4.88 [1.04 22.83] |  |  |  |  |  |  |  |  |  |
| Step 2 | BDI-II Score | .21 | .13 | 2.84 | .092 | 1.24 [.97 1.59] |  | 4.74 | .029 |  | 88.1 | 80.00 [51.91 95.67] | 92.59 [75.71 99.09] | 85.71 | 89.29 |
|  | Number of MDEs | 1.37 | .81 | 2.85 | .091 | 3.95 [.80 19.49] |  |  |  |  |  |  |  |  |  |
|  | F3/F4 avg. alpha | -1.32 | .67 | 3.95 | .047 | .27 [.07 .98] |  |  |  |  |  |  |  |  |  |
| Step 3 | BDI-II Score | .22 | .13 | 2.82 | .093 | 1.24 [.97 1.60] |  | .32 | .570 |  | 85.7 | 80.00 [51.91 95.67] | 88.89 [70.84 97.65] | 80.00 | 88.89 |
|  | Number of MDEs | 1.47 | .84 | 3.10 | .081 | 4.36 [.84 22.79] |  |  |  |  |  |  |  |  |  |
|  | F3/F4 avg. alpha | -1.25 | .68 | 3.39 | .066 | .29 [.08 1.08] |  |  |  |  |  |  |  |  |  |
|  | Sex | -.507 | .89 | .32 | .570 | .60 [.11 3.46] |  |  |  |  |  |  |  |  |  |
| * significant at *p* < .05 threshold, two-tailed. BDI-II, The Beck Depression Inventory II; Number of MDEs categorized into nonrecurrent [i.e., 1 previous episode], recurrent [2–4 episodes], and highly recurrent [≥5 episodes]. SE, standard error; CI, confidence interval; PPV, positive predictive value; NPV, negative predictive value. | | | | | | | | | | | | | | | |

# Supplementary Figures

**Supplementary Figure S1.** Exploratory comparison of alpha and theta power.

**
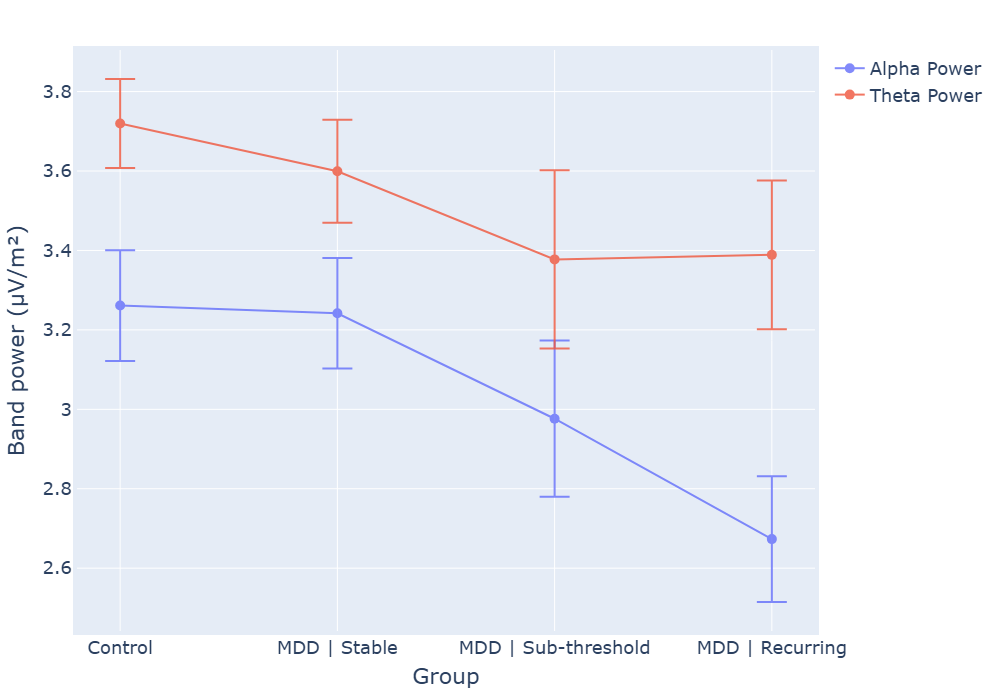
**

Depicted are averages and standard errors for alpha (8-13Hz) and theta (4-8Hz) power for each group at frontal electrodes F3 and F4 (averaged). See main manuscript Table 2.

**Supplementary Figure S2.** Alpha (8-13Hz) power at frontal electrodes (F3 and F4 averaged) for each group, split by sex. Errors bars show ± 1 standard error. Alpha power was calculated using CSD-transformed data.
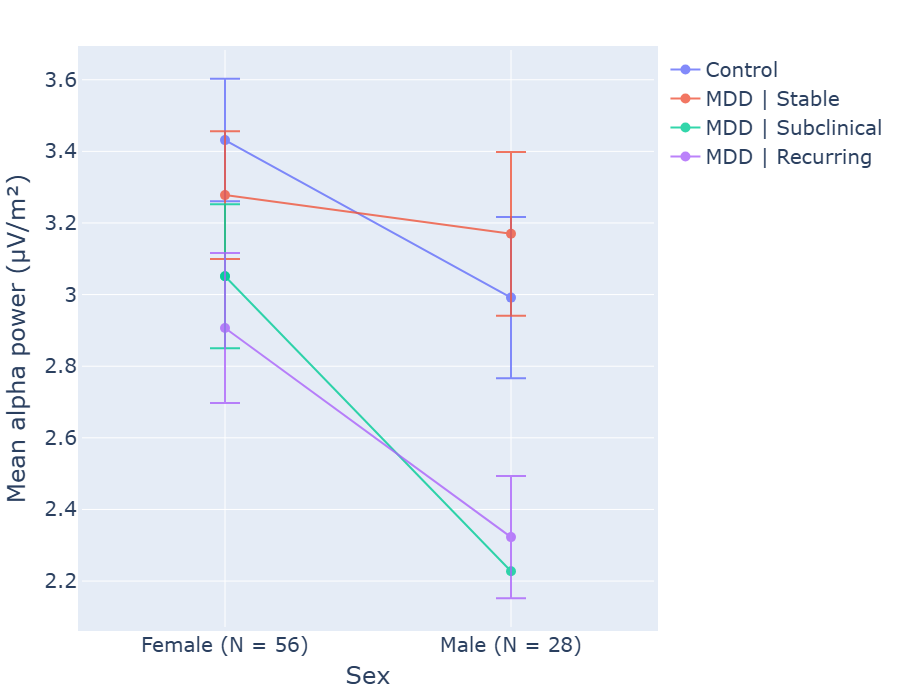


**Supplementary Figure S3 |** Results of K-means clustering of brain ICs across subjects. The three clusters depicted were chosen for having high levels of alpha power and greater projection to electrode sites F3 and F4, based on the scalp maps shown. Dipole locations for independent components included in each cluster are shown in blue. Dipole centroid locations are in yellow. MNI coordinates for dipole centroids are given. A | Cluster located in the left ventral anterior cingulate cortex. B | Cluster located in the right striatum. C | Cluster located in the left globus pallidus.


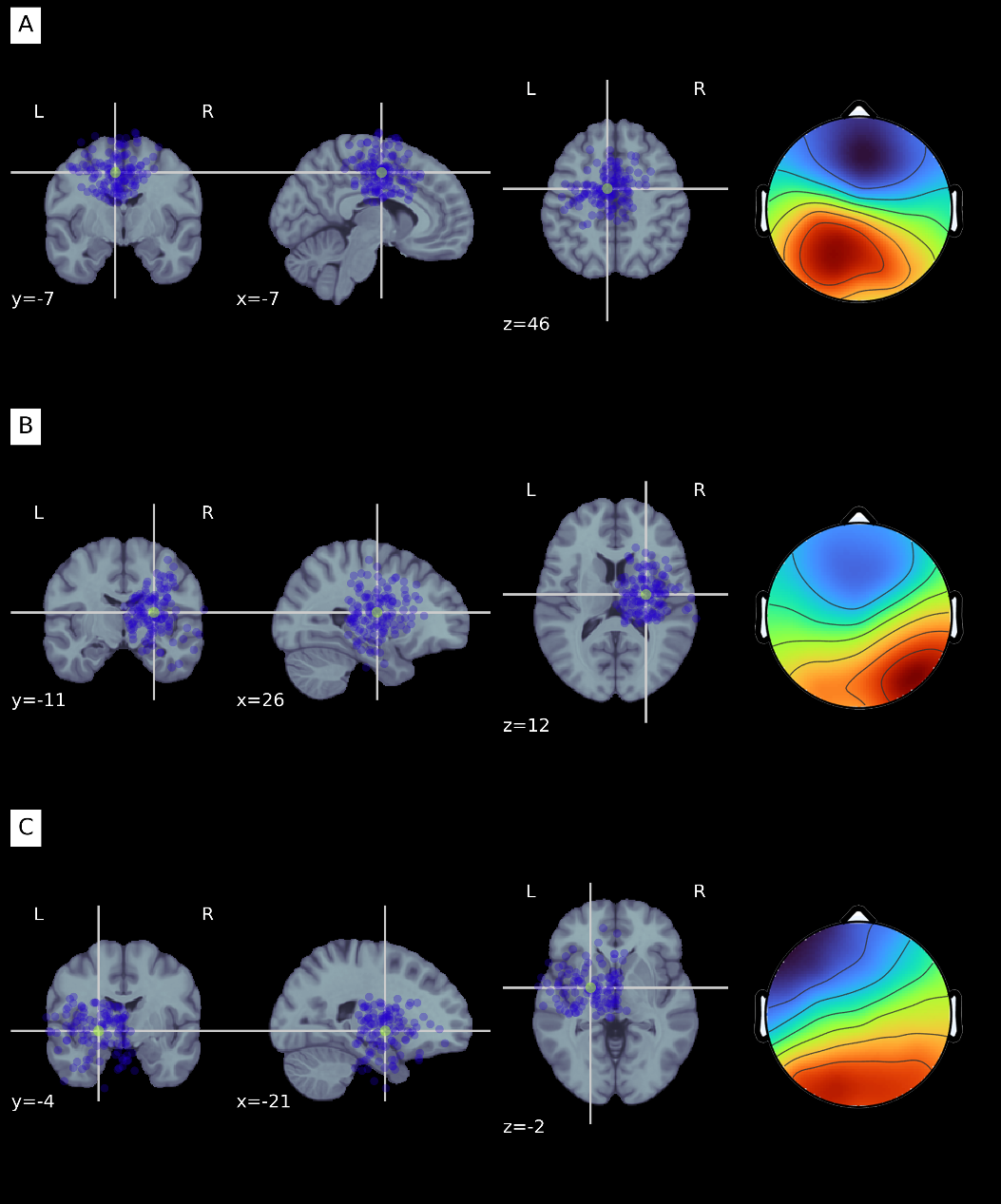

Supplement: Multimedia component 1 [file mmc1.docx]
